# The Effects of Different Thiol-Containing Compounds on the Degradation of Sulforaphene

**DOI:** 10.3390/molecules29184328

**Published:** 2024-09-12

**Authors:** Rui Gao, Pingxiang Liu, Jingxiu Bi, Yuying Jiang, Tong Zhao, Xuexia Yuan, Chao Zhang, Yutao Wang

**Affiliations:** 1 Shandong Provincial Key Laboratory of Test Technology on Food Quality and Safety, Institute of Quality Standard and Testing Technology for Agro-Products, Shandong Academy of Agricultural Sciences, Jinan 250100, China; 2 Cooperative of Vegetable and Grain Cultivation, Liaocheng Yifeng Bloc, Liaocheng 252000, China

**Keywords:** sulforaphene, thiol-containing compounds, degradation

## Abstract

Sulforaphene (4-methylsufinyl-3-butenyl isothiocyanate, SFE), produced by myrosinase hydrolysis of glucoraphenin (4-methylsulfinyl-3-butenyl glucosinolate) found in radish seeds, is strongly associated with cancer prevention. In this study, we investigated the stability of SFE (purity above 98%) under various thiol-containing compounds at 25 °C, such as sodium hydrosulfide (NaHS), glutathione (GSH), and cysteine (Cys). We observed that the degradation of SFE was closely related to the presence and dissociation capacity of thiol-containing compounds in the solution, particularly the thiol group. We found that the degradation rate of SFE was influenced by incubation with NaHS, GSH, and Cys, with distinct degradation products detected for each of these thiol-containing compounds. Compared to GSH, sulfide and Cys played important roles in promoting the degradation of SFE. Furthermore, we found substantial quantities of hydrogen sulfide in conjunction with SFE during the hydrolysis process of seeds, and a heat treatment of the seeds resulted in increased production of SFE. However, the introduction of sulfide-oxidizing bacteria to the hydrolytic system did not exhibit any inhibitory effect on the degradation of SFE. These results provided a guideline for industries to improve the stability of SFE during preparation.

## 1. Introduction

Some plant secondary metabolites, such as vinblastine, paclitaxel, and podophyllotoxin, have attracted considerable attention because of their anti-tumor effects and lower side effects than traditional radiotherapy and chemotherapy drugs [1,2]. Studies provide consistent evidence that the consumption of cruciferous vegetables, including radish, broccoli, cauliflower, and Chinese cabbage, could reduce the risk of many cancers and cardiovascular diseases [3,4,5,6], as these vegetables are rich in glucosinolates. Several pharmacological studies have indicated that the cancer prevention activity of cruciferous vegetables can be strictly attributed to isothiocyanates [7,8,9], which are derived from the rearrangement of the products hydrolyzed from glucosinolates by myrosinase (thioglucoside glucohydrolase, E.C. 3.2.3.1) [10]. Isothiocyanates are substances that give Brassica vegetables their pungent aroma and flavor, and they are responsible for the strong anticancer activity and anti-cardiovascular-disease properties [7,8,9].

SFE (4-methylsufinyl-3-butenyl isothiocyanate), a constituent of isothiocyanate family extracted from radish, has great potential in cancer prevention. It has been reported that SFE is capable of suppressing oesophageal squamous cell carcinoma growth by inhibiting MSK2 expression, in both human oesophageal squamous cells and PDX mouse model [11]. SFE could also inhibit the cell proliferation of endometrioid ovarian cancer [12], lung cancer cells [13], and human colon cancer cell lines HCT116, HT-29, KM12, SNU-1040, and DLD-1 [14] and suppress the migration and invasion of triple-negative SUM159 human breast cancer cells [15]. Moreover, SFE possesses neuroprotective [16] and anti-inflammatory [17] effects. These research has demonstrated that SFE possesses potential clinical utility as an effective cancer chemopreventive agent.

SFE is produced by the hydrolysis of glucoraphenin catalyzed by myrosinase, but SFE is unstable and easily degraded in aqueous solution, which impairs many of its useful applications [18]. Few studies have reported on the stability of SFE in various solvents and its degradation products. As reported by Yuan et al., the degradation product of SFE was identified to be 6-[(methylsulfinyl)methyl]-1,3-thiazinan-2-thione, based on the MS and NMR spectroscopy data [18]. SFE was sensitive to heat and could react with water to form a dimmer of SFE [19,20], and its -N=C=S group was also vulnerable to nucleophilic attacks of the hydroxyl groups in water, with –NH_2_ formed [20]. SFE was stable in acetonitrile, dichloromethane, ethyl acetate, or acetone, while alcohols could accelerate its degradation [19]. Moreover, sulforaphane was found to form N,N’-di-(methylsulfinyl) butyl thiourea in an aqueous solution [21]. Additionally, numerous studies have found that the electrophilic carbon atoms presented in isothiocyanate groups exhibited a tendency to undergo reactions with nucleophiles such as hydroxyl, amino, or thiol groups, resulting in the formation of o-thiocarbamates, thiourea derivants, or dithiocarbamates, respectively [22]. These results provide some references for studying the stability and degradation of SFE in solvents.

Given the ubiquitous presence of thiol compounds in living organisms, HS^−^, Cys, and GSH play crucial roles in diverse metabolic processes, potentially engaging in reactions with SFE during the hydrolytic process of radish seeds due to the high abundance of sulfur-containing secondary metabolites found in cruciferous vegetables [23]. The objective of this work was to study the influence of NaHS, Cys, and GSH on the stability of SFE in a PBS buffer at 25 °C. We found that Cys-SFE and HS^−^-SFE degradation products accounted for the largest proportion among all degradation products in the radish seeds hydrolytic process. We also found that the yield of SFE could increase after the heat-treatment of radish seeds. Based on the effects of sulfide, we tried to interfere SFE degradation by the addition of sulfide-oxidizing bacteria to the radish seeds’ hydrolytic system. These findings could provide a guideline and a new research direction for improving the stability of SFE during production and preparation.

## 2. Results and Discussion

### 2.1. SFE Analysis by High-Performance Liquid Chromatography (HPLC)

**Figure 1:**
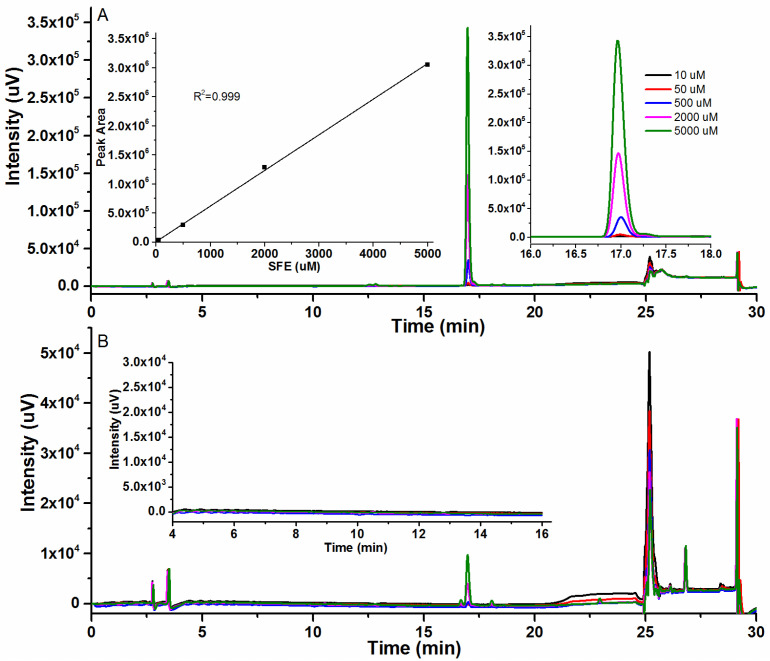
Chromatograms of SFE and its degradation product by HPLC. (**A**) Peak diagram of the SFE standard at 254 nm and its corresponding calibration curve. (**B**) Peak diagram of degradation products in SFE standard at 281 nm. SFE standard substance was dissolved into 10 μM, 50 μM, 500 μM, 2000 μM, and 5000 μM by acetonitrile, then directly injected to HPLC.

HPLC has proven to be an effective analytical tool for the detection of SFE and its degradation products. At 254 nm, HPLC allows us to identify the presence of SFE, while monitoring at 281 nm enables us to detect its degradation products. When different concentrations of SFE were analyzed at 254 nm, we could find that a conspicuous peak appeared at about 17.0 min, with no other obvious peak presented nearby, and the peak area or height at about 17.0 min was positively correlated with concentrations of SFE (Figure 1A). While the SFE standard was analyzed at 281 nm, except two small peaks appearing at 17.0 min, nearly no peaks appeared from 4 min to 20 min (Figure 1B). Typically, degradation products of SFE peak during this time period [18], indicating high purity and proper storage of the SFE standard. The results showed that SFE could be detected by HPLC at 254 nm, and there was no disturbance to the detection of SFE degradation products at 281 nm.

### 2.2. The Influence of Thiol-Containing Compounds on SFE

**Figure 2:**
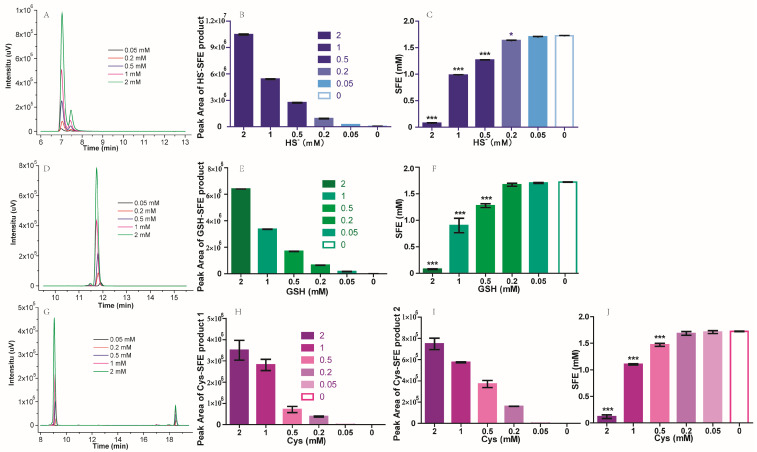
The influence of thiol-containing compounds on SFE. (**A**) Chromatograms of HS^−^-SFE degradation product at 281 nm. (**B**) The peak area of HS^−^-SFE product. (**C**) Residual content of SFE after reaction with NaHS at various concentrations. (**D**) Chromatograms of GSH^_^SFE product at 281 nm. (**E**) The peak area of GSH-SFE product. (**F**) Residual content of SFE after reaction with GSH at various concentrations. (**G**) Chromatograms of Cys^_^SFE product at 281 nm. (**H**) The peak area of Cys-SFE product 1 at 9.2 min. (**I**) The peak area of Cys-SFE product 2 at 18.8 min. (**J**) Residual content of SFE after reaction with Cys at various concentrations. Two mM SFE in PBS buffer reacted with 2 mM, 1 mM, 0.5 mM, 0.2 mM, and 0.05 mM thiol-containing compounds at 25 °C. After 1 h, the mixture was centrifuged and subjected into HPLC analysis system to analyze the contents of SFE and its degradation products. * *p* < 0.05; *** *p* < 0.001.

To investigate the influence of different thiol-containing compounds on SFE, NaHS, GSH, or Cys was added to 2 mM SFE standard in PBS buffer. Following a one-hour incubation at 25 °C, the resulting mixtures were subjected to HPLC analysis. Based on the chromatogram of HPLC, new peaks appeared at different retention times under 281 nm, after SFE reacted with NaHS, GSH, or Cys (Figure 2A,D,G). The peak of HS^−^-SFE reaction products appeared at about 7.0 min, and the peak of GSH-SFE reaction products appeared at about 11.8 min, while the Cys-SFE reaction products appeared at about 9.2 min and 18.8 min. We speculated the new peaks were the degradation products of SFE, as no peaks were detected for these thiol-containing compounds during this period. In addition, the concentration of thiol-containing compounds was positively correlated with an increase in their peak area, when the concentration of SFE remained constant. (Figure 2B,E–I). Moreover, a reciprocal relationship was observed between the peak areas of these new degradation products and that of SFE itself; as one intensifies, the other diminishes accordingly. The presence of different peaks and varying retention times suggested that multiple degradation products were formed depending on which thiol-containing compound was present. To confirm their specific identities, further verification is required through preparative liquid chromatography separation followed by mass spectrometry.

To find out the stoichiometric relationships between SFE and each thiol-containing compound, we carried out an analysis by reacting varying concentrations of thiol-containing compounds with a consistent concentration of SFE. As depicted in Figure 2C, the addition of 2 mM NaHS to 2 mM SFE resulted in an almost negligible detection of SFE, whereas a concentration of approximately 1 mM SFE was observed when the NaHS concentration was reduced to 1 mM. The stoichiometric relationship between SFE and NaHS was approximately 1:1. When the concentration of NaHS was below 0.2 mM, it had minimal impact on SFE. Cys and GSH exhibited a similar behavior to NaHS (Figure 2F,G). To consume 2 mM SFE, an equivalent amount of Cys or GSH was required. GSH is a tripeptide composed of glutamic acid (Glu), Cys, and glycine (Gly). Among these components, Glu is an acidic amino acid, and previous studies have reported that an acidic environment could inhibit the degradation of SFE [18]. Only the thiol group on Cys is susceptible to attack by SFE. Hence, in theory, the quantities of Cys and GSH required to consume an equivalent amount of SFE should be identical. These results were consistent with those studies’ conclusions that isothiocyanate groups has a tendency to react with thiol groups [18,22].

The correlation experiment was successfully replicated in the aprotic solvent acetonitrile, revealing no discernible degradation of SFE. One possible explanation was the stability of SFE in aprotic solvents [19], while another reason could be attributed to the challenging dissolution of the thiol group from NaHS, Cys, or GSH in acetonitrile.

### 2.3. The Generation of SFE in Radish Seeds’ Hydrolytic Process

**Figure 3:**
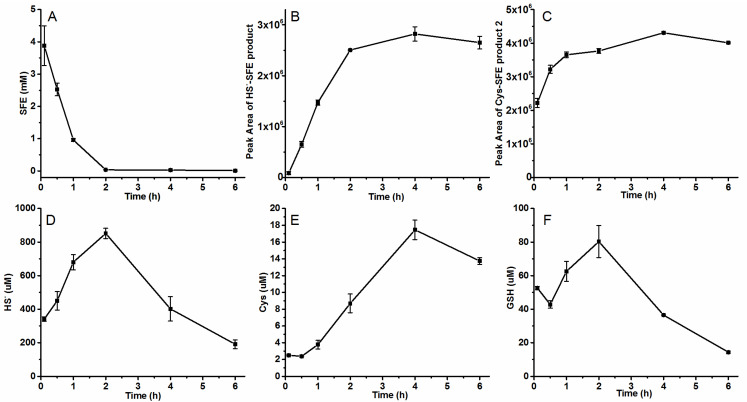
The concentration of each substance in radish seeds hydrolytic process. (**A**–**C**) the concentrations of SFE, HS^−^-SFE product, and Cys-SFE product 2 detected by HPLC analysis with PDA detector; (**D**–**F**) HS^−^, Cys, and GSH assayed by HPLC after mBBr derivatization with fluorescence detector. The radish seeds’ powders and PBS buffer were added to a screw-plugged tube at a ratio of 1:10, and the mixture was reacted at 25 °C. At different time intervals, 1 mL of the mixture were taken, centrifuged, and filtered with 0.22 μm filter. Part of the filtrate was used to detect HS^−^, Cys, and GSH after mBBr derivatization, and others was injected to HPLC to analyze the contents of SFE and its partial degradation products.

The main source of SFE is biological extraction, which involves hydrolyzing glucosinolates from broccoli or radish seeds [24,25,26]. During the hydrolysis process of radish seeds, we observed a decrease in SFE concentration from about 4 mM to 0.1 mM within 2 h (Figure 3A). This decline suggested that some substances which promote the degradation of SFE were produced during the hydrolytic process. Brassicaceae plants are abundant in GSH, sulfur-containing proteins, and Cys, which could be metabolized to produce sulfide (HS^−^) [27,28]. This result was consistent with the findings published by Song et al. [18], where SFE degraded within 120 min in the radish seed hydrolysis system, and the degradation product was formed when SFE was attacked by thiol groups and identified to be 6-[(methylsulfinyl)methyl]-1,3-thiazinan-2-thione.

We observed the corresponding peaks of HS^−^-SFE and Cys^_^SFE products (Figure 3B,C). The peak area of each degradation product increased when the SFE content decreased. By comparing the peak area in Figure 2, we found the amount of produced HS^−^-SFE at 4 h was equivalent to the products corresponding to 0.5 mM HS^−^. In contrast to in vitro experiments where Cys^_^SFE product 1 (9.2 min) was identified as the main degradation product, seed hydrolysis resulted in Cys^_^SFE product 2 (18.8 min) being the actual predominant product (Figure 3C). The peak area of Cys^_^SFE product 2 at 0.5 h reached a value of 3 × 10^6^, which exceeded the amount obtained from in vitro reaction products using a concentration of 2 mM Cys and SFE; moreover, the total amount mentioned was 4 times higher than that shown in Figure 2I for in vitro reaction products with similar concentrations of Cys and SFE. Although the peak area of produced GSH-SFE degradation and Cys^_^SFE product 1 increased with time, their overall numerical value remained relatively low (Figure S1). These results indicated that sulfides and cysteine were the main factors affecting the degradation of SFE.

Meanwhile, the HS^−^, Cys, and GSH produced in the hydrolytic process were detected. As degradation occurred, a large amount of HS^−^ was quickly produced in the seeds, providing sufficient thiols for reaction with SFE. The increase in HS^−^ was accompanied by the decrease in SFE and the increase in the formation of HS^−^-SFE product. Approximately 0.9 mM HS^−^ was detected two hours after hydrolysis, followed by a gradual decline in its concentration (Figure 3D). Thus, it could be inferred that the generation of HS^−^ in radish seeds was induced by SFE degradation. The correlation between SFE content and HS^−^ production suggested that HS^−^ played a crucial role in SFE degradation. Although there was generally an upward trend observed for Cys content, it remained at only a few micromoles (Figure 3E), as most Cys reacted with SFE. There were some changes in the content of GSH, but overall, it remained stable at low levels (0.2–1 μmol·g^−1^) (Figure 3F), which was similar to what has been reported for apple embryo axes isolated from seeds (0.3–0.7 μmol·g^−1^) [29]. Additionally, there may exist numerous other known (homocysteine) or unknown thiol containing compounds within it. Based on these findings, both HS^−^ and Cys exerted more influence on SFE degradation than GSH.

### 2.4. Effect of Sulfide-Oxidizing Bacteria Cupriavidus Pinatubonensis JMP134 on the SFE Degradation in Radish Seed Hydrolytic Process

Considering the substantial production of HS^−^ during radish seed hydrolysis, we aimed to mitigate SFE degradation by introducing enzymes capable of metabolizing the generated HS^−^. *C. pinatubonensis* JMP134, known for its ability to oxidize sulfide to sulfate using sulfide: quinone oxidoreductase (SQR) and persulfide dioxygenase (PDO), was employed in this study. Both wild-type and *sqr-pdo* knockout strains of *C. pinatubonensis* JMP134 were incorporated into the radish seed hydrolysis system to investigate their impact on SFE degradation.

**Figure 4:**
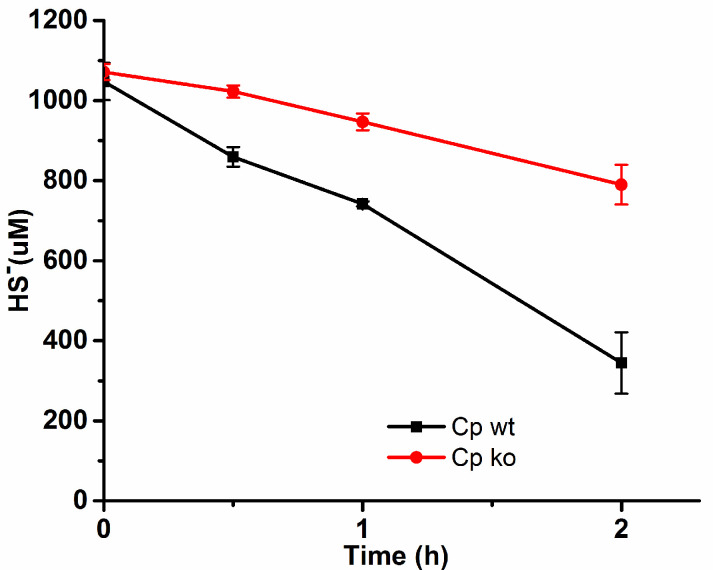
The detection of HS^−^ consumption. Cells were resuspended in 50 mM PBS buffer (pH 7.2) at an OD600 of 5 and disrupted. NaHS was added to 1.0 mM to initiate the reaction at 25 °C. Sulfide was tested at different time intervals by methylene blue method.

The cells were cultured, harvested, and resuspended in 50 mM PBS buffer at pH 7.4. About 1 mM NaHS was added to the suspension above to assess its HS^−^ consumption capacity. The wild-type of *C. pinatubonensis* JMP134 (Cp wt) rapidly oxidized 0.6 mM sulfide within a span of 2 h, whereas *sqr-pdo* knockout strains of *C. pinatubonensis* JMP134 (Cp ko) exhibited negligible sulfide oxidation capacity (Figure 4). These results demonstrated that this strain could be employed for subsequent experiments aimed at sulfide removal.

**Figure 5:**
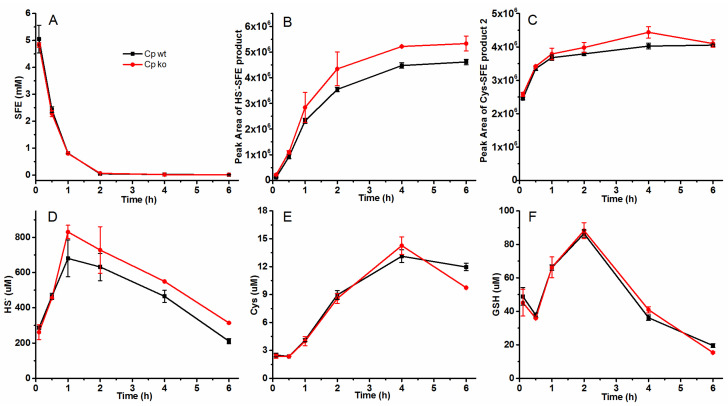
The concentration of each substance in radish seed hydrolytic process with or without sulfide-oxidizing bacteria. (**A**–**C**) the concentrations of SFE, HS^−^-SFE product, and Cys-SFE product 2 detected by HPLC analysis with PDA detector; (**D**–**F**) HS^−^, Cys, and GSH assayed by HPLC after mBBr derivatization with fluorescence detector. Ten mL OD_600_ = 5 Cp wt or Cp ko resuspension was added into 1 g seed powders, respectively. The reaction was carried out at 25 °C for 6 h. At different time intervals, 1 mL mixture were taken, centrifuged, and filtered with 0.22 μm filter. Part of the filtrate was used to detect HS^−^, Cys, and GSH after mBBr derivatization, and others were injected to HPLC to analyze the contents of SFE and its partial gradation products.

As shown in Figure 5, upon addition of the two strains to the radish seed hydrolysis system, there was no significant difference observed in both the concentration of SFE and the amount of Cys^_^SFE degradation (Figure 5A,C). However, the peak area of produced HS^−^-SFE degradation and the concentration of HS^−^ in the system was higher after 1 h when Cp ko was added (Figure 5C,D). Furthermore, no notable disparity was found between the concentrations of Cys and GSH (Figure 5E,F). Additionally, the peak area of produced GSH-SFE product and Cys^_^SFE product 1 (9.2 min) (Figure S2) was more than 10 times smaller than the amount obtained from in vitro reaction products using 1 mM GSH/ Cys with 2 mM SFE showed in Figure 2. The main degradations of SFE were also HS^−^-SFE and Cys^_^SFE product 2 (18.8 min).

The peak area of HS^−^-SFE product was equivalent to that of products resulting from the reaction between 1 mM HS^−^ and 2 mM SFE, while the peak area of Cys-SFE product 2 was over 5 times greater than that of products resulting from the reaction between 2 mM Cys reacted and 2 mM SFE. It seemed that Cys-SFE product 2 was dominant rather than HS^−^-SFE product. The addition of Cp wt could consume the HS^−^ produced during the radish seed hydrolysis but did not effectively inhibit SFE degradation.

### 2.5. Effect of Heat-Treatment on the SFE Degradation in Radish Seed Hydrolytic Process

**Figure 6:**
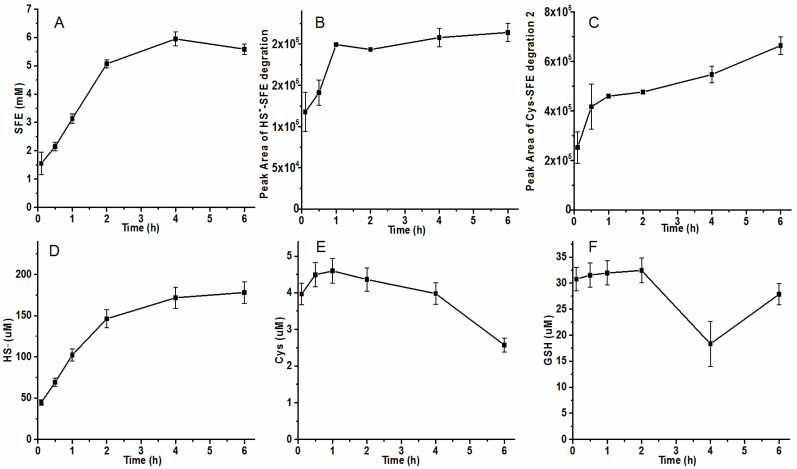
The concentration of each substance in the hydrolytic process of heat-treatment seeds. (**A**–**C**) the concentrations of SFE, HS^−^-SFE product, and Cys-SFE product 2 detected by HPLC analysis with PDA detector; (**D**–**F**) HS^−^, Cys, and GSH assayed by HPLC after mBBr derivatization with fluorescence detector. The seed powders and PBS buffer were added to a screw-plugged tube at a ratio of 1:10, and the mixture reacted at 25 °C. At different time intervals, 1 mL of the mixture was taken, centrifuged, and filtered with 0.22 μm filter. Part of the filtrate was used to detect HS^−^, Cys, and GSH after mBBr derivatization, and others were injected to HPLC to analyze the contents of SFE and its partial degradation products.

Radish seeds were heated for 1 h to deactivate some sulfide-produced enzymes like cystathionine-γ-lyase, cystathionine-β-synthase, or 3-mercapto-sulfurtransferase. Subsequently, the heat-treatment seeds were ground and hydrolyzed to determine the concentration of SFE, HS^−^, Cys, and GSH. By hydrolyzing heat-treated seeds, there was an accumulation trend observed in SFE content with a sustained level of 5 mM after 2 h and nearly no degradation over the subsequent two hours (Figure 6A). The initial concentration of SFE was relatively low, possibly due to the inhibition or inactivation of certain myrosinase upon heating. Additionally, little HS^−^-SFE was detected (Figure 6B). After a half hour hydrolysis period of the radish seeds, the amount of Cys-SFE product 2 corresponded well with that obtained from a reaction containing 0.5 mM Cys and 2 mM SFE reaction (Figure 6C). Meanwhile, both GSH-SFE and Cys-SFE product 1 still remained at very low levels (Figure S3). At the same time, the concentrations of produced HS^−^, Cys, and GSH were lower than unheated seeds (Figure 6D–F). The reduced production of HS^−^-SFE product during the hydrolytic process in heat-treated seeds may be attributed to decreased sulfide generation by sulfide-producing enzymes.

We also used a paper strip with lead(II)-acetate to detect produced HS^−^ by observing lead(II)-sulfide black precipitates [30]. There was little blackening observed in heat-treated seeds’ hydrolytic process after 6 h, while visible black at the bottom of the strip was seen in the untreated seeds’ hydrolytic process, although they both produced large amounts of black precipitates over longer hydrolysis times (Figure S4).

## 3. Materials and Methods

### 3.1. Materials

Standard substance SFE (purity > 98%) was purchased from Chengdu Derick Biotechnology Co., Ltd. (Chengdu, China). Radish seeds were obtained from Jiangxi Ganxin Seed Co., Ltd. (Nanchang, China). Methanol, acetonitrile, acetic acid, and trifluoroacetic acid (TFA) used for analytical HPLC were of chromatographic grade and purchased from Thermo Fisher Scientific Inc. (Tustin, CA, USA). Ultra-pure water was obtained by Q Millipore System (Millipore, France). NaHS (purity > 70%), GSH (purity 98%), and cysteine hydrochloride (purity 99%) were purchased from Sangon Biotech Co., Ltd. (Shanghai, China). Monobromodimane (mBBr) was purchased from MedChemExpress LLC (Shanghai, China). Other chemicals such as Lysogeny broth (LB), phosphate-buffer saline (PBS), and dimethyl sulfoxide(DMSO) were purchased from Sinopharm Chemical Reagent Co., Ltd. (Shanghai, China).

### 3.2. HPLC Analysis

SFE and its degradation products were tested by a reversed-phase HPLC system with a C18 column (250 mm × 4.6 mm, 5 μm; Shimadzu, Kyoto, Japan) referring to Yuan et al.’s method with slight modifications [18]. Briefly, the mobile phase system consisted of methanol as mobile phase A and 0.02% (*v*/*v*) TFA aqueous solution as mobile phase B. The flow rate was 1 mL/min, the injection volume was 10 μL, and the column temperature was maintained at 40 °C. The detection wavelengths were 254 nm and 281 nm. The column was eluted with the following gradients of mobile phase B: 6–60% from 0 to 22 min; 60–100% from 22 to 23 min; 100% from 23 to 26 min; 100 to 6% from 26 to 27 min; 6% from 27 to 30 min.

NaHS, Cys, and GSH were detected after mBBr derivatization by using HPLC, according to published methods [31,32] with minor modifications. Briefly, 30 μL of 1 M Tris-base and 10 μL of 25 mM mBBr were added to 100 μL supernatants, which was mixed and incubated for 20 min at room temperature in the dark. An equal volume of acetonitrile and acetic acid mixture (9:1) was added. After mixing, the sample was centrifuged at 15,000× *g* for 2 min, and 10 μL of the supernatant was injected into HPLC. A reverse phase C18 column (250 mm × 4.6 mm, 5 μm; Shimadzu, Kyoto, Japan) was pre-equilibrated with 80% mobile phase A (0.25% acetic acid) and 20% mobile phase B (90% methanol and 0.25% acetic acid) and maintained at 30 °C. The column was eluted with the following gradients of mobile phase B: 8–60% from 0 to 1 min; 60–100% from 1 to 15 min; 100% from 15 to 20 min; 100 to 8% from 20 to 21 min; and 8% from 21 to 31 min. The flow rate was 0.8 mL/min. The derivants were detected by a fluorescence detector with excitation at 370 nm and emission at 485 nm.

### 3.3. The Influence of Thiol-Containing Compounds on the Stability of SFE

SFE was dissolved into 22.8 mM by acetonitrile as the storage solution. The reactions took place in the PBS buffer with an initial SFE concentration of 2 mM. Different thiol-containing compounds were added into the above solution to each concentration. At the same time, an equal volume of buffer was added as a negative control. The mixture was allowed to spontaneously react for different time intervals at 25 °C. Then, the sample of the mixture was filtered with 0.22 μm filter and subjected to the HPLC analysis system to analyze the contents of SFE and its degradation products.

### 3.4. The Radish Seed Hydrolysis Process

The radish seeds were ground into powders with an analytical grinder. The heat-treatment radish seeds were heated at 100 °C for one hour in a drying oven and then ground. One g powders were added to 10 mL PBS buffer. The mixture was allowed to spontaneously hydrolyze for different time intervals at 25 °C. When needed, thiol-containing compounds or cell suspensions were added to the hydrolysis system. The mixture was filtered with 0.22 μm filter after being centrifuged at 12,000 rpm/min for 5 min. Then, the generated SFE was analyzed by HPLC.

### 3.5. Preparation of Cell Suspension and Detection of Sulfide HS^−^

*C. pinatubonensis* JMP134 (Cp wt) and its *sqr-pdo* knockout strain (Cp ko) were cultivated in LB at 30 °C with shaking. Cells were harvested by centrifugation, resuspended in 50 mM PBS buffer at pH 7.4 to an OD600 of 5. About 1 mM NaHS was added to the suspension to detect its HS^−^ consumption capacity. Sulfide was detected with a diamine reagent [33].

The method used for the detection of H_2_S production was the same as described by Xia et al. [30]. Briefly, add radish seeds powders and PBS buffer to a screw-plugged tube at a ratio of 1:10, and a paper strip with lead(II)-acetate was affixed at the top of the tube with a screw plug. The tubes were incubated at 25 °C for 6 h and 48 h, and then, the paper strips were photographed to detect any black precipitates of lead(II)-sulfide, as an indicator of H_2_S production.

### 3.6. Data Processing and Statistical Analysis

All analyses were repeated three times, and the data were expressed as means and standard deviations. Analysis was carried out by using Origin 2015 and GraphPad Prism version 5. Data were expressed as mean values ± standard deviations, and differences between groups were evaluated by using the One-way ANOVA analysis.

## 4. Conclusions

In this study, we found a correlation between the degradation of SFE and the presence of the thiol group, as well as the thiol dissociation capacity of compounds in the solution. The degradation products varied depending on specific thiol-containing compounds that attacked SFE. Notably, sulfide and Cys exhibited the most significant impact on SFE, suggesting that reducing their production could effectively enhance SFE yield. Although the addition of sulfide-oxidizing bacteria *C. pinatubonensis* JMP134 to the hydrolytic system of radish seeds could reduce sulfide concentration level, it had no effect on inhibiting the degradation of SFE. Conversely, hydrolyzing heat-treated radish seeds could result in the higher yield of SFE. SFE plays important physiological roles, but its instability, easy degradation, and complicated synthesis features limit its manufacture. Therefore, further exploration is warranted to identify additional methods for slowing down or inhibiting its degradation.

## Data Availability

The original contributions presented in the study are included in the article and Supplementary Material, further inquiries can be directed to the corresponding authors.

## Supplementary Materials

The following supporting information can be downloaded at: https://www.mdpi.com/article/10.3390/molecules29184328/s1, Figure S1: GSH-SFE product (A) and Cys-SFE product 1 (B) in radish seeds hydrolytic process; Figure S2: GSH-SFE product (A) and Cys-SFE product 1 (B) in radish seeds hydrolytic process in radish seed hydrolytic process with or without sulfide-oxidizing bacteria; Figure S3: GSH-SFE product (A) and Cys-SFE product 1 (B) in hydrolytic process of heat-treatment seeds; Figure S4: Production of hydrogen sulfide during hydrolytic process of heated radish seeds and untreated seeds.

## References

[1] Dholwani K.K., Saluja A.K., Gupta A.R., Shah D.R. (2008). A review on plant-derived natural products and their analogs with anti-tumor activity. Indian J. Pharmacol..

[2] Motyka S., Jafernik K., Ekiert H., Sharifi-Rad J., Calina D., Al-Omari B., Szopa A., Cho W.C. (2023). Podophyllotoxin and its derivatives: Potential anticancer agents of natural origin in cancer chemotherapy. Biomed. Pharmacother..

[3] Abdull Razis A.F., Noor N.M. (2013). Cruciferous vegetables: Dietary phytochemicals for cancer prevention. Asian Pac. J. Cancer Prev..

[4] Ren H.G., Luu H.N., Liu Y., Wang D.W., Guo X. (2023). High intake of cruciferous vegetables reduces the risk of gastrointestinal cancers: Results from observational studies. Crit. Rev. Food Sci. Nutr..

[5] Cardozo L.F.M.F., Alvarenga L.A., Ribeiro M., Dai L., Shiels P.G., Stenvinkel P., Lindholm B., Mafra D. (2021). Cruciferous vegetables: Rationale for exploring potential salutary effects of sulforaphane-rich foods in patients with chronic kidney disease. Nutr. Rev..

[6] Ağagündüz D., Şahin T.Ö., Yılmaz B., Ekenci K.D., Duyar Özer Ş., Capasso R. (2022). Cruciferous vegetables and their bioactive metabolites: From prevention to novel therapies of colorectal cancer. Evid. Based Complement. Altern. Med.

[7] Abbaoui B., Lucas C.R., Riedl K.M., Clinton S.K., Mortazavi A. (2018). Cruciferous vegetables, isothiocyanates, and bladder cancer prevention. Mol. Nutr. Food Res..

[8] Hudlikar R., Wang L., Wu R., Li S., Peter R., Shannar A., Chou P.J., Liu X., Liu Z., Kuo H.-C.D. (2020). Epigenetics/epigenomics and prevention of early stages of cancer by isothiocyanates. Cancer Prev. Res..

[9] Na G., He C., Zhang S., Tian S., Bao Y., Shan Y. (2023). Dietary isothiocyanates: Novel insights into the potential for cancer prevention and therapy. Int. J. Mol. Sci..

[10] Bhat R., Vyas D. (2019). Myrosinase: Insights on structural, catalytic, regulatory, and environmental interactions. Crit. Rev. Biotechnol..

[11] Zhang C., Wu Q., Yao K., Jin G., Zhao S., Zhang J., Zheng W., Xu e., Zu Y., Yuan J. (2023). Sulforaphene suppresses oesophageal cancer growth through mitogen- and stress-activated kinase 2 in a PDX mouse model. Am. J. Cancer Res..

[12] Yu H.Y., Yang L., Liu Y.C., Yu A.J. (2023). Sulforaphene suppressed cell proliferation and promoted apoptosis of COV362 cells in endometrioid ovarian cancer. PeerJ.

[13] Yang M., Wang H., Zhou M., Liu W., Kuang P., Liang H., Yuan Q. (2016). The natural compound sulforaphene, as a novel anticancer reagent, targeting PI3K-AKT signaling pathway in lung cancer. Oncotarget.

[14] Byun S., Shin S.H., Park J., Lim S., Lee E., Lee C., Sung D., Farrand L., Lee S.R., Kim K.H. (2016). Sulforaphene suppresses growth of colon cancer-derived tumors via induction of glutathione depletion and microtubule depolymerization. Mol. Nutr. Food Res..

[15] Bao C., Kim M.C., Chen J., Song J., Ko H.W., Lee H.J. (2016). Sulforaphene interferes with Human breast cancer cell migration and invasion through inhibition of hedgehog signaling. J. Agric. Food Chem..

[16] Yang W., Liu Y., Xu Q.-Q., Xian Y.-F., Lin Z.-X. (2020). Sulforaphene ameliorates neuroinflammation and hyperphosphorylated tau protein via regulating the PI3K/Akt/GSK-3 β pathway in experimental models of Alzheimer’s disease. Oxidative Med. Cell. Longev..

[17] Hwang H.J., Kim J.-E., Lee K.W. (2022). Sulforaphene attenuates *Cutibacterium acnes*-induced inflammation. J. Microbiol. Biotechnol..

[18] Song D., Liang H., Kuang P., Tang P., Hu G., Yuan Q. (2013). Instability and structural change of 4-methylsulfinyl-3-butenyl isothiocyanate in the hydrolytic process. J. Agric. Food Chem..

[19] Tian G., Tang P., Xie R., Cheng L., Yuan Q., Hu J. (2016). The stability and degradation mechanism of sulforaphene in solvents. Food Chem..

[20] Tian G., Li Y., Cheng L., Yuan Q., Tang P., Kuang P., Hu J. (2016). The mechanism of sulforaphene degradation to different water contents. Food Chem..

[21] Jin Y., Wang M., Rosen R.T., Ho C.T. (1999). Thermal degradation of sulforaphane in aqueous solution. J. Agric. Food Chem..

[22] Zhang Y., Talalay P. (1994). Anticarcinogenic activities of organic isothiocyanates: Chemistry and mechanisms. Cancer Res..

[23] Bonnot T., Bachelet F., Boudet J., Le Signor C., Bancel E., Vernoud V., Ravel C., Gallardo K. (2023). Sulfur in determining seed protein composition: Present understanding of its interaction with abiotic stresses and future directions. J. Exp. Bot..

[24] Kuang P., Song D., Yuan Q., Yi R., Lv X., Liang H. (2013). Separation and purification of sulforaphene from radish seeds using macroporous resin and preparative high-performance liquid chromatography. Food Chem..

[25] Wang L., Jiang H., Liang X., Zhou W., Qiu Y., Xue C., Sun J., Mao X. (2021). Preparation of sulforaphene from Radish seed extracts with recombinant food-grade *Yarrowia lipolytica* harboring high myrosinase activity. J. Agric. Food Chem..

[26] Liu Y., Zhang D., Li X., Xiao J., Guo L. (2022). Enhancement of ultrasound-assisted extraction of sulforaphane from broccoli seeds via the application of microwave pretreatment. Ultrason. Sonochem..

[27] Fuentes-Lara L.O., Medrano-Macias J., Perez-Labrada F., Rivas-Martinez E.N., Garcia-Enciso E.L., Gonzalez-Morales S., Juarez-Maldonado A., Rincon-Sanchez F., Benavides-Mendoza A. (2019). From elemental sulfur to hydrogen sulfide in agricultural soils and plants. Molecules.

[28] Ito T., Ohkama-Ohtsu N. (2023). Degradation of glutathione and glutathione conjugates in plants. J. Exp. Bot..

[29] Ciacka K., Tyminski M., Gniazdowska A., Krasuska U. (2022). Cold stratification-induced dormancy removal in apple (*Malus domestica* Borkh.) seeds is accompanied by an increased glutathione pool in embryonic axes. J. Plant Physiol..

[30] Xia Y., Lu C., Hou N., Xin Y., Liu J., Liu H., Xun L. (2017). Sulfide production and oxidation by heterotrophic bacteria under aerobic conditions. ISME J..

[31] Newton G.L., Dorian R., Fahey R.C. (1981). Analysis of biological thiols: Derivatization with monobromobimane and separation by reverse-phase high-performance liquid chromatography. Anal. Biochem..

[32] Xin Y., Liu H., Cui F., Liu H., Xun L. (2016). Recombinant *Escherichia coli* with sulfide:quinone oxidoreductase and persulfide dioxygenase rapidly oxidises sulfide to sulfite and thiosulfate via a new pathway. Environ. Microbiol..

[33] Cline J.D. (1969). Spectrophotometric determination of hydrogen sulfide in natural waters. Limnol. Ocean..

