# Supplementary material for: The Effects of Different Thiol-Containing Compounds on the Degradation of Sulforaphene
Source: Molecules. 2024 Sep 12;29(18):4328. doi: 10.3390/molecules29184328 (PMC11434082; doi:10.3390/molecules29184328)
Supplement: Supplementary file 1 [file molecules-29-04328-s001.zip › molecules-3149507-supplementary.pdf]

Supporting information

The Effects of Different Thiol-Containing Compounds on the Degradation of Sulforaphene

Rui Gao[1], Pingxiang Liu[1], Jingxiu Bi[1], Yuying Jiang[1], Tong Zhao[1], Xuexia Yan[1], Chao Zhang[1], and Yutao Wang[1,2,*]

Content:

Figure S1—Figure S4

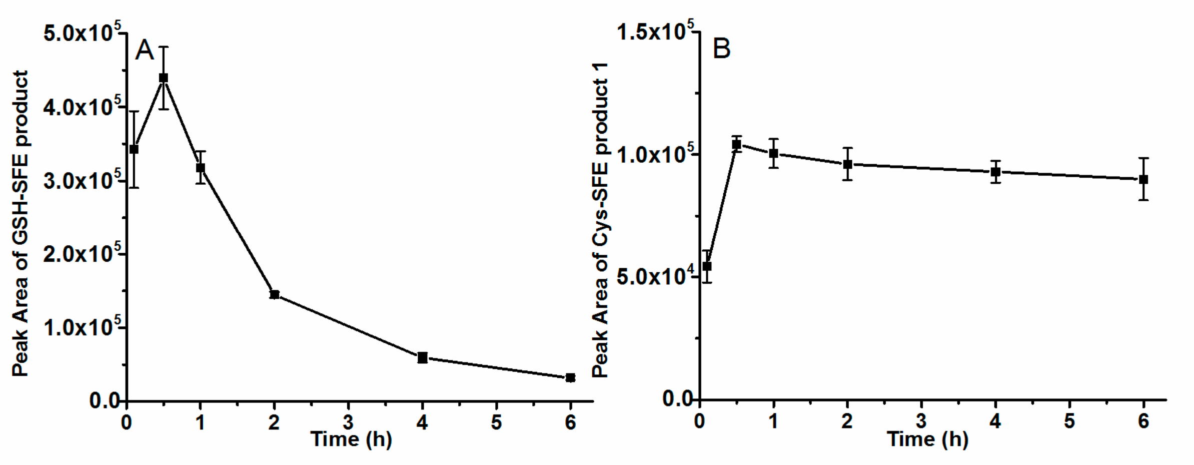

Figure S1. GSH-SFE product (A) and Cys-SFE product 1 (B) in radish seeds hydrolytic process. The radish seeds powders and PBS buffer were added to screw-plugged tube at a ratio of 1:10, the mixture was reacted at 25°C. At different time intervals, 1mL of the mixture were taken, centrifuged, filtered with 0.22 μm filter and injected to HPLC.

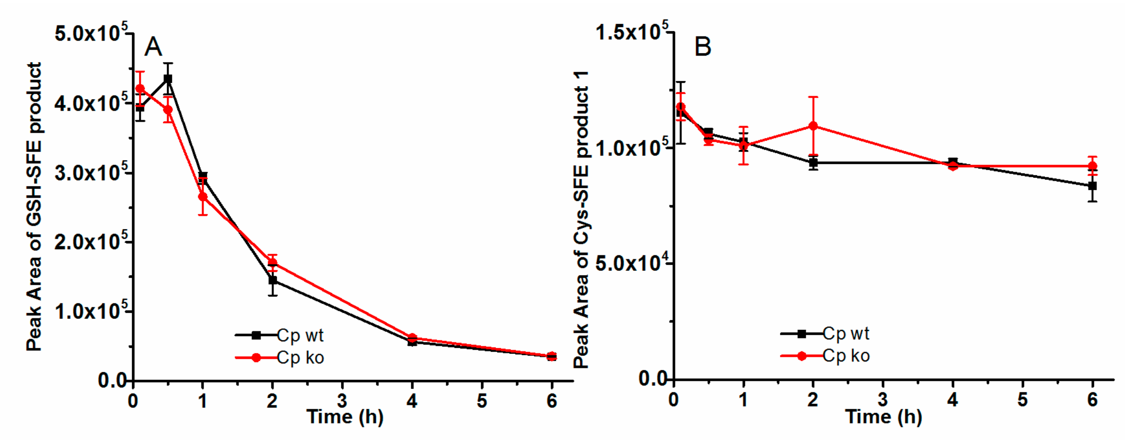

Figure S2. GSH-SFE product (A) and Cys-SFE product 1 (B) in radish seeds hydrolytic process with or without sulfide oxidizing bacteria. 10 mL $OD_{600} = 5$ Cp wt or Cp ko resuspension was added into 1 g seed powders, respectively. The reaction was carried out at 25°C for 6 h. At different time intervals, 1mL of the mixture were taken, centrifuged, filtered with 0.22 μm filter and injected to HPLC.

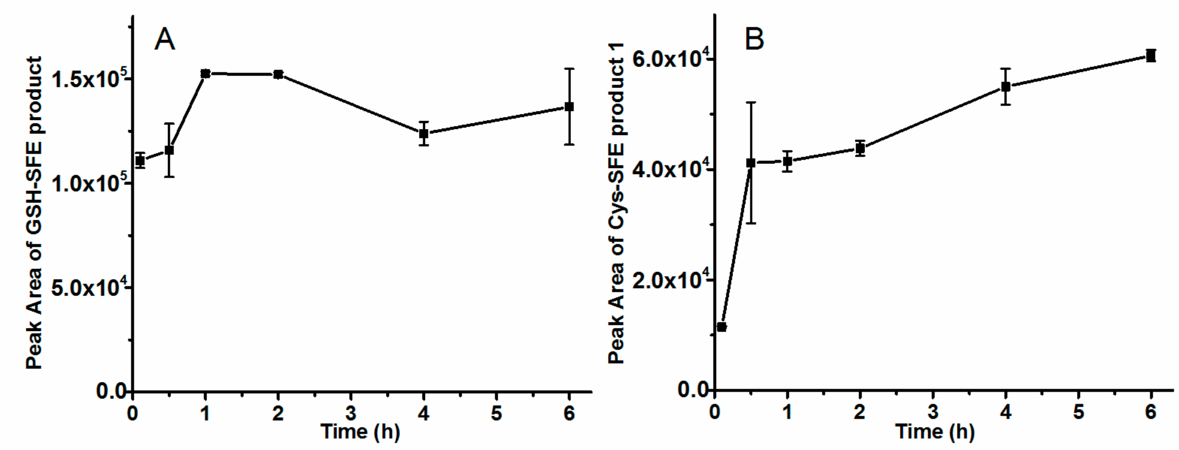

Figure S3. GSH-SFE product (A) and Cys-SFE product 1 (B) in hydrolytic process of heat-treatment seeds. The heated radish seeds powders and PBS buffer were added to screw-plugged tube at a ratio of 1:10, the mixture was reacted at 25°C. At different time intervals, 1mL of the mixture were taken, centrifuged, filtered with 0.22 μm filter and injected to HPLC.

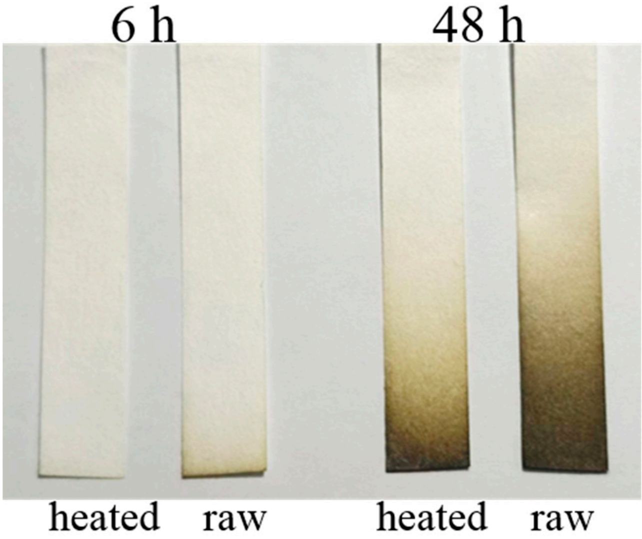

Figure S4. Production of hydrogen sulfide during hydrolytic process of heated radish seeds and untreated seeds.
